# Abnormal Presentation of an Intra-abdominal Desmoid Tumor in a Young, Healthy Male: A Case Report

**DOI:** 10.7759/cureus.114877

**Published:** 2026-08-20

**Authors:** Erica Kozorosky, Konstantina Kostara, Zohaa Ahmad, Benetta Miller, Zbigniew Moszczynski

**Affiliations:** 1 General Surgery, CarePoint Health, Bayonne, USA; 2 General Surgery, Kansas City University, Kansas City, USA

**Keywords:** cystic necrosis, desmoid, intra-abdominal desmoid, mesenteric tumor, pathogenesis

## Abstract

Desmoid tumors are soft tissue proliferations with a tendency to invade surrounding tissues and, when occurring in the mesentery, can present with obstructive symptoms, pain, or organ damage. This report presents a case of a 33-year-old male with no medical history or contributory family history who developed systemic infectious symptoms related to cystic necrosis of a desmoid tumor. The cystic necrosis suggests that the tumor may have grown so rapidly that it outgrew its vascular supply before causing any symptoms, highlighting the diverse range of presentations and emphasizing individualized treatment plans.

## Introduction

Desmoid tumors of the mesentery, also known as desmoid fibromatosis or aggressive fibromatosis, are a rare type of fibroblastic proliferation distinguished by their ability to develop in soft tissues, their infiltrative nature, and their tendency to recur [1,2]. Globally, desmoid tumors account for two to four cases per one million people annually, with a female-to-male ratio of 3:2 and a median age at diagnosis of 30 to 40 years [3]. Mechanisms underlying the predominance of desmoid cases diagnosed in women and younger individuals include hormonal influences such as estrogen, genetic diseases involving the adenomatous polyposis coli (APC) gene, such as familial adenomatous polyposis or Gardner’s syndrome, and events such as prior surgery or trauma [3-5]. Furthermore, desmoid tumors of the mesentery specifically can be classified as extra-abdominal or intra-abdominal, and some may even cause symptoms such as bowel obstruction, pain, or organ damage [6]. Current treatment protocols include surveillance or observation if asymptomatic, whereas surgery is considered if patients are symptomatic and if there is functional impairment due to the desmoid tumor [7].

Herein, we present a rare case of an intra-abdominal desmoid tumor in a 33-year-old male with no history of prior trauma or familial adenomatous polyposis, successfully treated with surgical excision.

## Case presentation

**Figure 1:**
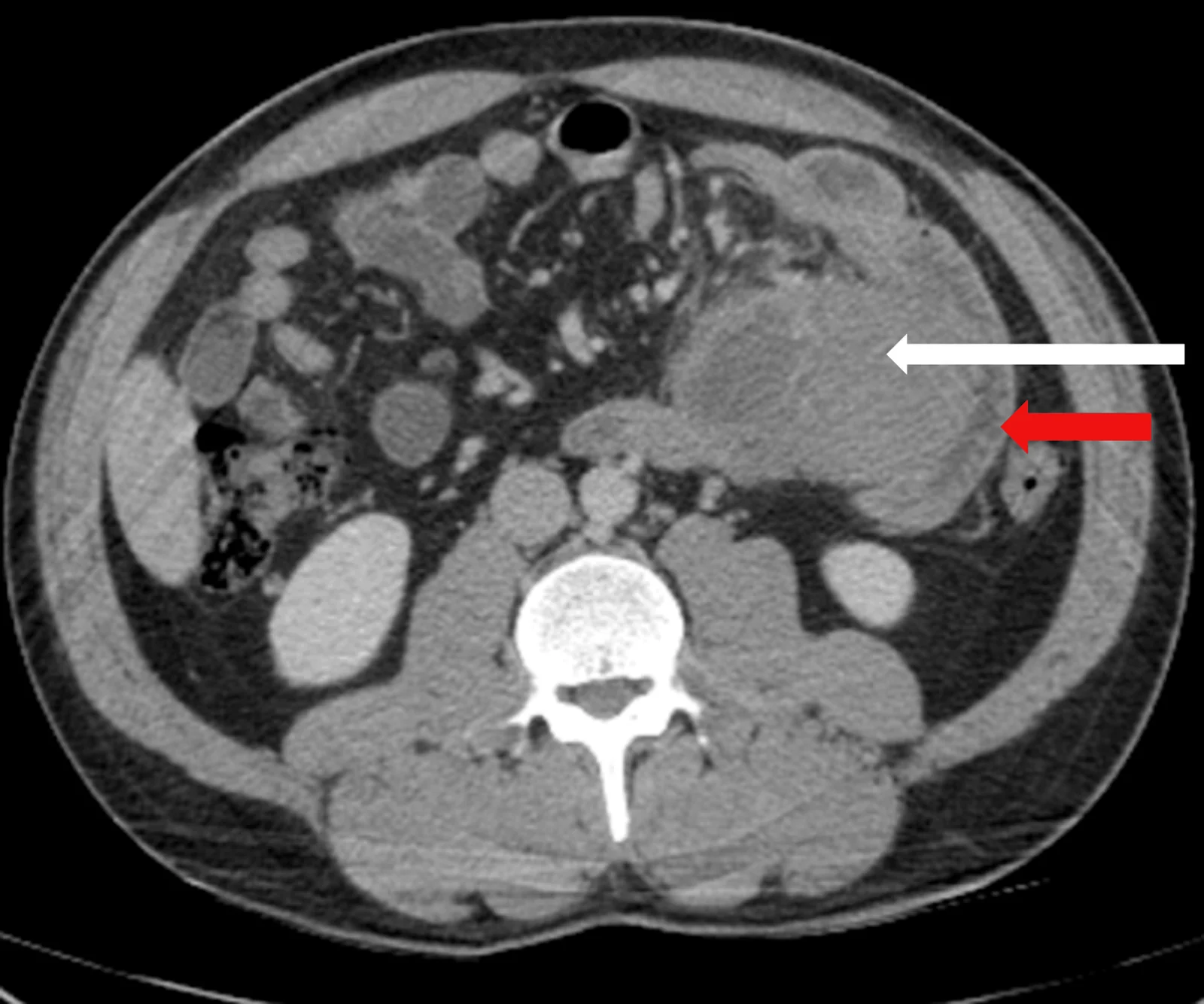
Axial CT image demonstrating a heterogeneous mass (white arrow) within the mesentery, displacing adjacent jejunal loops (red arrow)

**Figure 2:**
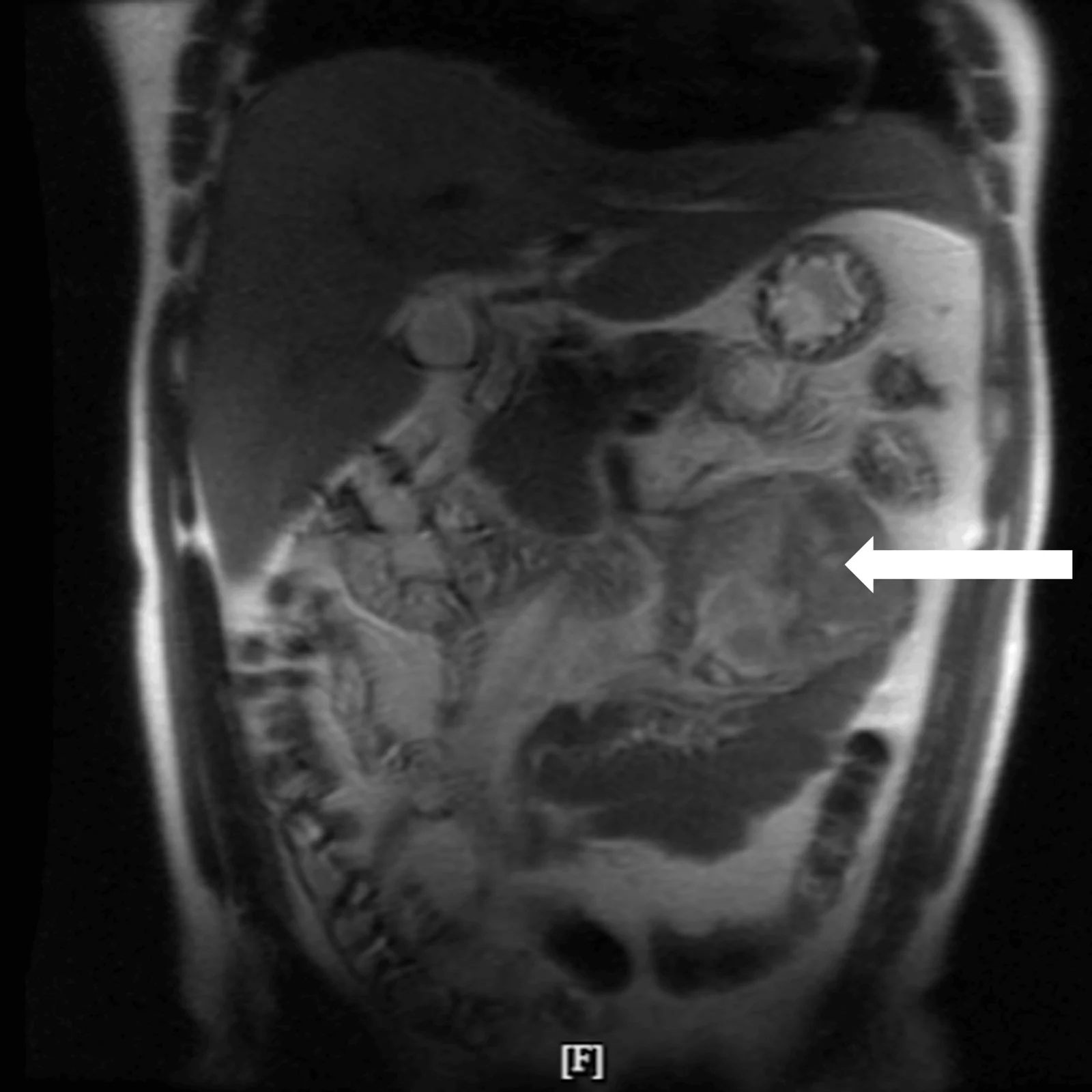
Coronal MRI image demonstrating a heterogeneously enhancing mixed cystic-solid lesion (white arrow) within the mesentery, abutting the small bowel

The patient is a 33-year-old male with no significant past medical or surgical history who presented to the emergency department with four days of progressively worsening diffuse abdominal pain, abdominal distension, low-grade fever, and decreased stool output. He denied nausea, vomiting, hematochezia, or recent sick contacts, travel, or dietary changes. On arrival, he was febrile to 101.4°F, tachycardic to 108 bpm, and had leukocytosis of 13,000 cells/μL (Tables 1, 2). Initial CT of the abdomen and pelvis revealed a large, heterogeneous, left-sided mesenteric mass displacing adjacent jejunal loops, with no evidence of obstruction, which was new compared with a CT performed two years prior (Figure 1). MRI confirmed an 8.3 × 6.4 × 8.1 cm well-circumscribed, heterogeneous, enhancing mass with central cystic changes, closely associated with the small bowel (Figure 2).

**Table 1:** Vital signs at presentation, indicating a fever and tachycardia MAP: mean arterial pressure; SpO₂: peripheral oxygen saturation

Vital sign	Result	Unit	Reference range
Temperature	101.4	°F	97.0-99.5 °F
Temperature source	Oral	-	-
Heart rate	108	beats/min (bpm)	60-100 bpm
Blood pressure	126/60	mmHg	<120/<80 mmHg
MAP	82	mmHg	70-100 mmHg
Respiratory rate	20	breaths/min	12-20 breaths/min
SpO₂	98	%	95-100%
Oxygen delivery	Room air	-	-

**Table 2:** Labs at presentation, indicating leukocytosis WBC: white blood cell; RBC: red blood cell; Hgb: hemoglobin; Hct: hematocrit; MCV: mean corpuscular volume; MCH: mean corpuscular hemoglobin; MCHC: mean corpuscular hemoglobin concentration; RDW: red cell distribution width; MPV: mean platelet volume; MDW: monocyte distribution width

Laboratory test	Result	Unit	Reference range
WBC count	13	×10³/µL	4.0-11.0
RBC count	4.73	×10⁶/µL	4.20-5.80
Hgb	13.3	g/dL	12.0-17.5
Hct	39.8	%	36-53
MCV	84.1	fL	80-100
MCH	28.1	pg	27-33
MCHC	33.4	g/dL	32-36
RDW	13.1	%	11.5-14.5
Platelet count	326	×10³/µL	150-450
MPV	7	fL	7.5-11.5
Neutrophils	68	%	40-70
Lymphocytes	16	%	20-40
Monocytes	14.7	%	2-8
Eosinophils	0.9	%	0-5
Basophils	0.4	%	0-1
Absolute neutrophil count	8.9	×10³/µL	1.5-8.0
Absolute lymphocyte count	2.1	×10³/µL	1.0-4.0
Absolute monocyte count	1.9	×10³/µL	0.2-0.8
Absolute eosinophil count	0.1	×10³/µL	0.0-0.5
Absolute basophil count	0	×10³/µL	0.0-0.2
MDW	20.81	-	<20.0

**Figure 3:**
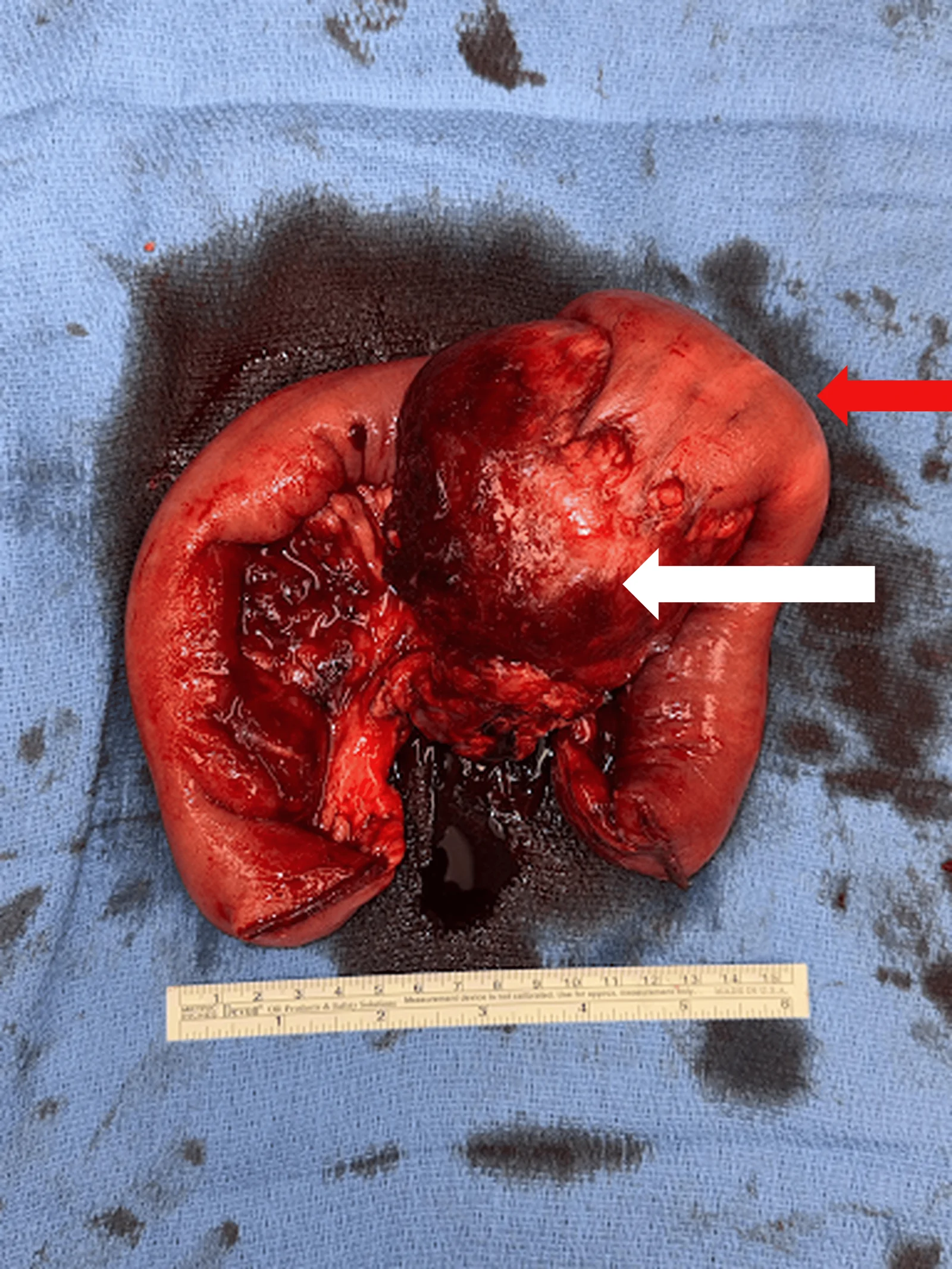
Gross excised specimen demonstrating a mesenteric mass with cystic components and focal necrosis (white arrow). The adjacent small bowel wall appears intact and distinct from the mass (red arrow)

The patient was started on broad-spectrum antibiotics and evaluated by general surgery, gastroenterology, hematology-oncology, and infectious disease. Blood cultures remained negative. Persistent fevers were noted, and stool cultures were sent, which were also negative for any infectious pathogens. Given the persistent symptoms and imaging findings, the decision was made to take the patient to the operating room. An exploratory laparotomy with en bloc resection of the mesenteric mass, small bowel resection with side-to-side primary anastomosis, and abdominal washout was performed. Intraoperative findings included an 8 × 10 cm soft tissue mass involving the mesentery of the proximal jejunum, just distal to the ligament of Treitz, with a perforation at the tip and purulent spillage, although the bowel wall was intact (Figure 3).

Postoperatively, antibiotics were continued per infectious disease recommendations, with cultures of the abdominal fluid growing Citrobacter and Klebsiella. The patient recovered well postoperatively, with stable hemodynamics, an afebrile status, return of bowel function, and tolerance of diet advancement. The patient was evaluated by gastroenterology, who advised the patient to follow up for an outpatient colonoscopy, and oncology, who elected to follow up after the pathology results were available. The pathology report identified the mass as desmoid fibromatosis. The small bowel margins were negative for disease, and all four harvested lymph nodes were negative for tumor cells. Immunostaining was performed at an outside facility, which showed that the cells stained positive for beta-catenin and weakly positive for SMA. Negative staining for desmin, S100, c-kit, DOG1, and MUC4 ruled out leiomyoma, gastrointestinal stromal tumor (GIST), and fibromyxoid sarcoma, respectively.

The patient followed up for repeat imaging six months postoperatively, which showed no evidence of recurrence. Follow-up with gastroenterology and oncology is still pending.

## Discussion

Desmoid tumors are locally invasive tumors with mesenchymal features, accounting for approximately 0.03% of soft tissue neoplasms [8,9]. Sporadic mutations in the Wnt/β-catenin pathway account for 90% of cases, whereas approximately 10% of cases are associated with APC gene mutations, manifesting in 10%-30% of patients with FAP [8,10]. In addition to genetic predisposition, estrogen is hypothesized to play a role in the pathogenesis of desmoid tumors [3-5].

Specifically, in one study by Migmeni et al., estrogen receptor beta was observed in all desmoid tumor samples, suggesting that androgens modulate the Wnt/β-catenin pathway implicated in the pathogenesis of desmoid tumors [11]. Prior trauma is another common association with the development of desmoid tumors, most likely due to the initiation of the wound-healing cascade. During wound healing, fibroblasts are activated by two pathways: the Wnt/β-catenin pathway and the Notch pathway. Normally, the Wnt pathway is terminated following repair, leading to the degradation of β-catenin and arrest of fibroblast proliferation. However, in cases where there is a mutation in the CTNNB1 gene, which plays a role in the Wnt/β-catenin pathway, this process may be disrupted [12].

Clinically, desmoid tumors present as slow-growing masses, with approximately 20% of cases resulting in mass regression [8]. While some patients present asymptomatically, desmoid tumors are associated with decreased quality of life due to the high symptom burden of this disease process [6]. In one literature review analyzing the incidence of symptoms in patients with desmoid tumors, up to 63% of patients presented with pain, followed by limited range of motion, weakness, and fatigue [6]. Moreover, other commonly noted symptoms include bowel obstruction or perforation, as well as hydronephrosis, secondary to the rapid proliferation and obstructive nature of these tumors [13].

Our patient presented with fever, leukocytosis, and progressively worsening abdominal pain, a presentation that is rare compared with the traditional obstruction-related symptoms in most desmoid tumor cases. Furthermore, intraoperative findings of a soft tissue mass involving the mesentery of the proximal jejunum, with a perforation at the tip and purulent spillage and an intact bowel wall, along with positive abdominal cultures for Citrobacter and Klebsiella, suggest that the infectious symptoms were caused by a perforation secondary to the desmoid tumor, more rarely occurring without evidence of bowel obstruction.

Our case mirrors that of Li et al., in which an 18-year-old female presented with abdominal pain, nausea, vomiting, and leukocytosis [14]. CT imaging demonstrated a 7.1 × 6.7 × 5.9 cm mass in the right lower quadrant, along with small intestine compression and free intraperitoneal air; however, there was no bowel dilatation [14]. Upon resection of the mass, which was found to be adherent to the ileum, 300 mL of pus was present, as well as perforation of the specimen, indicating that the patient’s symptoms were a result of intestinal perforation caused by the desmoid tumor [14]. However, it is important to note that the patient had undergone an appendectomy two years prior to the presentation of symptoms, whereas our patient did not have any previous history of abdominal surgery or trauma.

Given the rare and unique presentation of desmoid tumors, it is important to emphasize the need for individualized management. Most cases of desmoid tumors begin with a tumor growth phase and are followed by a period of growth arrest [6]. In patients who are asymptomatic, surveillance is considered sufficient [15]. However, in patients who are either symptomatic or show rapid growth, surgical resection may be warranted; medical therapy is an alternative for patients who are either poor surgical candidates or for whom surgical resection is not ideal [15]. In our patient, there was a large amount of purulent fluid within the mass, suggesting that it outgrew its vascular supply and began to necrose. The indication of rapid proliferation, along with the symptomatic features of the disease, ultimately led to resection of the desmoid tumor. As illustrated by this case, desmoid tumors present on a clinical spectrum and thus require a range of treatment options, from conservative measures to rapid interventions, highlighting the importance of an individualized approach.

## Conclusions

Mesenteric desmoid tumors are rare, locally aggressive neoplasms with variable presentations, ranging from asymptomatic incidental findings on abdominal imaging to bowel obstruction and perforation. This case demonstrates an atypical presentation in a patient who presented with systemic infectious symptoms due to central necrosis rather than the classic obstructive symptoms, despite the large size of the tumor. While desmoid tumors do not typically have malignant potential, this case illustrates how they can exhibit rapid local expansion and vascular compromise, reinforcing the importance of individualized management.
